# Hidden syndinian and perkinsid infections in dinoflagellate hosts revealed by single-cell transcriptomics

**DOI:** 10.1093/ismejo/wrae188

**Published:** 2024-09-26

**Authors:** Elizabeth C Cooney, Brian S Leander, Patrick J Keeling

**Affiliations:** Department of Botany, University of British Columbia, Vancouver, BC V6T 1Z4, Canada; Department of Botany, University of British Columbia, Vancouver, BC V6T 1Z4, Canada; Department of Zoology, University of British Columbia, Vancouver, BC V6T 1Z4, Canada; Department of Botany, University of British Columbia, Vancouver, BC V6T 1Z4, Canada

**Keywords:** MALV, dinoflagellate, single-cell transcriptomics, parasite, Amoebophrya, perkinsid

## Abstract

Free-living core dinoflagellates are commonly infected by members of two parasitic clades that are themselves closely related to dinoflagellates, the marine alveolates and perkinsids. These parasites are abundant and ecologically important, but most species have been difficult to observe directly or cultivate, so our knowledge of them is usually restricted to environmental 18S rRNA gene sequences, as genome-scale molecular data are not available for most species. Here, we report the finding of several of these parasites infecting free-living dinoflagellates. Of the 14 infected host cells collected, only five were identified as containing parasites via light microscopy at the time of collection. Single-cell transcriptome sequencing yielded relatively high transcriptomic coverage for parasites as well as their hosts. Host and parasite homologs were distinguished phylogenetically, allowing us to infer a robust phylogenomic tree based on 192 genes. The tree showed one parasite belongs to an undescribed lineage that is sister to perkinsids, whereas the remainder are members of the syndinian clade within the marine alveolates. Close relatives of all these parasites have been observed in 18S rRNA gene surveys, but until now none had been linked to a specific host. These findings illustrate the efficacy of single-cell isolation and transcriptome sequencing as strategies for gaining deeper insights into the evolutionary history and host relationships of hidden single-celled parasites.

## Introduction

In dinoflagellates and close relatives, parasitism has arisen multiple times, resulting in many independent lineages that infect diverse protist and metazoan hosts [1]. While parasitic dinoflagellates have long been documented [2], it was not until the use of environmental 18S rRNA gene sequencing that their depth of diversity and potential ecological impact became clear: not only are these parasites globally distributed, but in the oceans they account for several of the most abundant eukaryotic lineages known [3, 4].

Phylogenies have revealed that one important parasitic group, the marine alveolates (MALVs), is composed of at least five distinct subgroups [5] that cluster into two major lineages: MALVs II and IV (the Syndiniales, or “syndinians”), and MALVs I, III, and V [1]. Despite accounting for up to 50% of eukaryotic sequences in environmental samples and being thought to be the dominant parasitic group within the picoplanktonic size fraction [6–9], visual observations of most MALV species are scant or non-existent, as they are likely tiny in their free-living stages and difficult to detect when inside a host. Another major subgroup of parasites branching sister to dinoflagellates, the perkinsids, are less abundant in environmental samples but more widely distributed, as they are also common in terrestrial and freshwater ecosystems [10, 11].

Both MALVs and perkinsids infect a wide variety of host animals and protists, including the closely related core dinoflagellates [12–14]. In particular, *Amoebophrya* and other members of the syndinian MALV II clade [5] infect many free-living dinoflagellates, including toxic species, contributing so significantly to bloom depletion that they surpass zooplankton grazers in some cases [15, 16]. For this reason, *Amoebophrya* is relatively well studied, but little is known about host-specificity or the diversity of its hosts, because most dinoflagellates are not in culture and early stages of infection are not always visible in cells observed in the field.

While conducting a large single-cell transcriptome sequencing survey to investigate the molecular diversity of uncultured marine dinoflagellates, we discovered that a subset of the cells we collected (14 in total out of 168 cells analyzed) were infected by syndinian MALV IIs or perkinsids (Table S1). Hosts were a range of core dinoflagellates, including photosynthetic and heterotrophic species, as well as thecate and athecate species. Uninfected individuals of most host species were also collected, or in a few cases already had transcriptome data available, giving us paired infected/uninfected datasets. We performed a multi-protein phylogenomic analysis on all cells by searching for 263 conserved genes in each transcriptome (Table S2) and generating trees for all genes [17]. For each gene, host and parasite orthologs (and contamination) were differentiated based on phylogenetic affinities to closely related species included in the alignments. This approach allowed us to resolve the phylogenomic position of parasites independent of their hosts. For further details of this methodology, see Supplementary Methods.

**Figure 1:**
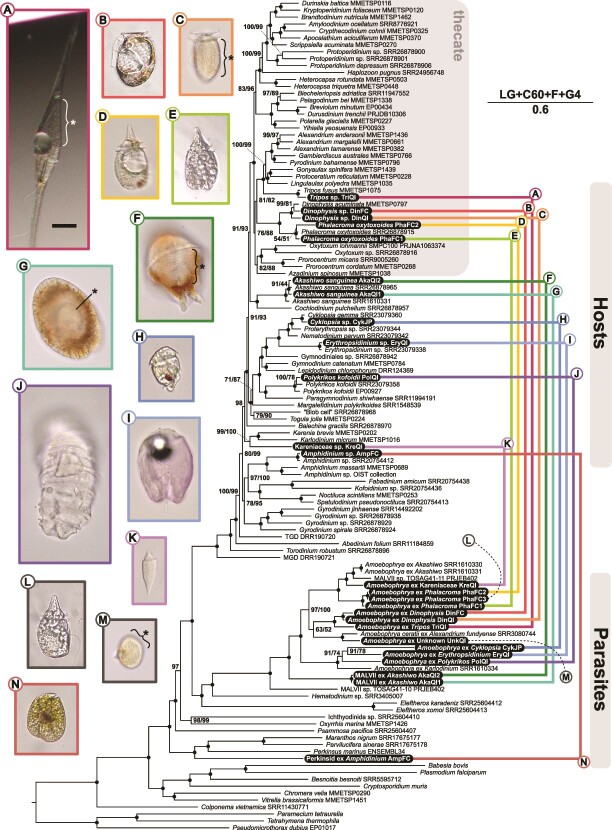
Maximum likelihood phylogenomic tree of parasites and their core dinoflagellate hosts. The primary model used to generate this tree from 192 conserved gene alignments is shown with a scale bar representing the estimated number of amino acid substitutions per site. Node numbers indicate bootstrap values ([LG + C60 + F + G4 − bb 1000]/[LG + C60 + F + G4 PMSF −b 100]); black dots signify 100 for both analyses. Transcriptomes introduced in this study are highlighted in black. Host and parasite pairs are connected by colored lines. Cells in light micrographs are all depicted at the same scale; scale bar = 25 μm. For cells with infections that were identifiable at the time of collection, parasites are indicated by asterisks.

**Figure 2:**
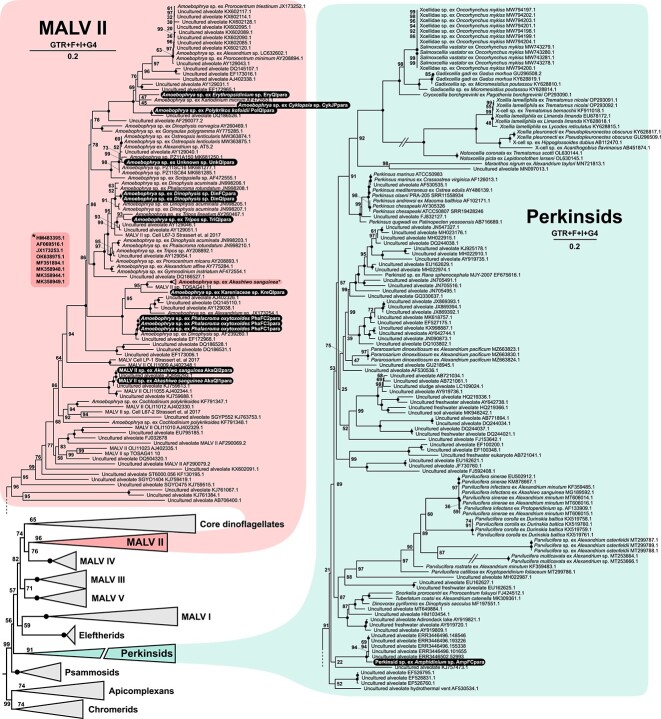
Maximum likelihood phylogeny inferred from 18S rRNA gene sequences showing the diversity of syndinian MALV II and perkinsid parasites. The model used to infer the tree and the scale bar representing the estimated number of nucleotide substitutions per site are shown next to each clade. Node numbers indicate bootstrap values, with black dots signifying 100. Taxa introduced in this study are highlighted in black. Broken branches are depicted at half their original length.

The multi-gene phylogeny revealed that all but one parasite clustered with the monophyletic MALV II clade within the syndinians, with the single exception being an infection of *Amphidinium* sp. that branched sister to the clade containing *Maranthos*, *Parvilucifera*, and *Perkinsus* (Fig. 1). In the same tree, hosts cluster closely with members of the same species, with the exception of KreQI, for which transcriptomes of the same species are not available. For two cells (PhaFC3, UnkQI), host transcripts were very sparse and were thus omitted from this analysis. We also tested these relationships using a phylogeny of 18S rRNA gene sequences, which allows for a much more diverse collection of both cultured and environmentally derived sequences from many more lineages of early-diverging dinoflagellates, including all MALV groups and perkinsids (Fig. 2).

The 18S rRNA gene phylogeny revealed that the perkinsid relative did not share a close phylogenetic affinity with any currently classified group within the perkinsid clade (i.e. *Perkinsus*, *parviluciferans*, and *Xcellidae*). Instead, it grouped with a clade of environmental sequences branching outside the known perkinsids and was most closely related to sequences retrieved from benthic environments [1]. At the time of collection, there was no visual indication that the marine *Amphidinium* sp. host cell was infected, other than a red-pigmented area in the center, which has not been observed in non-infected cells of the same species [18]. This ambiguity and the paucity of highly similar sequences in environmental sampling suggest this species is rare and unlikely to be found without extensive molecular screening of potential host species.

All other parasites were confirmed to belong to the syndinian MALV II clade in the 18S rRNA gene phylogeny (Fig. 2). This also suggests that most of these parasites belong to the genus *Amoebophrya*, because they all branch with clades designated as such; however, the two identical parasites found to infect *Akashiwo sanguinea* branched outside the well-annotated *Amoebophrya* clade, so we refer to them simply as “MALV II.” These cells fell within a clade of environmental sequences, all of which lacked information about their potential hosts. *A. sanguinea* has already been shown to be infected by an *Amoebophrya* species that is genetically distinct (Fig. 2; [19]), so this is the second member of the MALV II clade now confirmed to infect this host. The 18S rRNA gene sequences of most *Amoebophrya* sp. collected here shared phylogenetic affinities with existing sequences of parasites infecting closely related hosts. Those infecting *Cyklopsia*, *Erythropsidinium*, and *Polykrikos*, all unarmored cells belonging to the order *Gymnodiniales*, branched adjacent to one another.

Infections by perkinsids and syndinians that have been studied in any detail lead to host cell death, often relatively quickly. Some of these parasites are easily found due to identifiable signs of infection (e.g. the beehive stage of MALV II or the germ tube in some perkinsids [20]), but for undescribed species of these groups, it is unknown whether we can distinguish infected from uninfected hosts in live samples, as even well-known parasites are nearly invisible during most life stages. Although approaches like environmental sample incubation have proven effective for growing and observing different life stages of some parasite species, these methods are likely not viable for much of their diversity, since many host species, including some in this study, perish hours after collection.

The assemblage of parasites we present here, many of them hidden, demonstrates the efficacy of single-cell isolation and transcriptome sequencing for gaining molecular insights into diverse, uncultured parasites. Although extricating host and parasite transcriptomes can pose a challenge, this method generates a depth of genetic information for parasitic single-celled eukaryotes that can be parsed for robust phylogenomic and functional analysis, unambiguously linking parasites to a host species, and does not require *a priori* knowledge of the host–parasite interaction. These are all valuable criteria to better understand a major ecological dynamic that is very difficult to study in the absence of cultured systems.

## Supplementary Material

Supplementary_Methods_wrae188

Table_S1_wrae188

Table_S2_wrae188

## Data Availability

The data underlying this article are available in the article, in the Supplementary Information online, and on Genbank (PP905692-PP905717; SRA: PRJNA1121905). Nucleotide and translated amino acid assemblies and videos are at https://doi.org/10.5683/SP3/8BIRAL.
