# Supplementary material for: Hidden syndinian and perkinsid infections in dinoflagellate hosts revealed by single-cell transcriptomics
Source: ISME J. 2024 Sep 26;18(1):wrae188. doi: 10.1093/ismejo/wrae188 (PMC11468006; doi:10.1093/ismejo/wrae188)
Supplement: Supplementary_Methods_wrae188 [file supplementary_methods_wrae188.docx]

**Supplementary Methods**

**Single cell collection and imaging**

Cells in the present study were among 168 cells collected as part of a larger effort to sequence interesting and understudied dinoflagellates in the coastal waters of British Columbia, Canada [1–4]. Marine samples were gathered using a plankton net (20 µm mesh) deployed from public docks at the mouth of Snauq (also known as False Creek; 49.277010, -123.139921), from Jericho Pier (49.276996, -123.201612) in Vancouver, and from shore at the Hakai facility on the north side of Quadra Island (50.115097, -125.220766). Once collected, net tow samples were refrigerated at 4°C and scrutinized for dinoflagellates under a Leica DM IL LED microscope as soon as possible, usually within 2 hours of when the sample was taken. All cells in the present study were collected from July 2020 to September 2021. Dates and locations for each infected cell can be found in Table S1.

Some cells in this study were isolated because they showed visible signs of infection, but most were collected with the assumption they were healthy individuals of the host species, collected because they represented lineages for which transcriptomic datasets were not yet available. Cells were isolated using a glass microcapillary pipet, washed in 0.2 µm syringe filtered sea water from the source sample, and imaged with a Sony alpha 7III, before being placed into lysis buffer [5]; videos (only available for some cells) are available at https://doi.org/10.5683/SP3/8BIRAL. Samples were immediately frozen and stored at -70°C until extraction.

**Sequencing and transcriptome assembly**

Single cell samples were processed according to the Smart-seq2 protocol to produce Illumina cDNA libraries [5, 6]. Nextera Flex libraries were prepared from cDNA by the Sequencing and Bioinformatics Consortium at University of British Columbia and sequenced on the Nextseq (Illumina) platform. Adapters and primers were trimmed from forward and reverse raw reads using Cutadapt [7] before assembly with rnaSPAdes v3.15.1 [8]. Decontamination was performed on assemblies by searching NCBI nt and Uniprot reference proteomes [9] with Blastx and Blastn [10] respectively, followed by the identification and removal of contaminant contigs using BlobTools [11]. Decontamination was performed uniformly on all assemblies, removing identifiable bacterial, metazoan, fungal, chlorophyte, haptophyte, and diatom contigs. Amino acid sequences were predicted from nucleotide assemblies by identifying and annotating open reading frames with TransDecoder v5.5.0 [12] and a BlastP search (e-value threshold ≤1e10^-5^) against the Uniprot database [13]. Estimations of completeness of assembled amino acid transcriptomes were generated using BUSCO v5.4.3 [14], comparing against the alveolate_odb10 database (Table S1).

**Identification and phylogenomic analysis**

Many infected cells showed no visible signs of infection and were collected with the assumption that they were healthy individuals. After assembly, 18S rDNA sequences were extracted from each transcriptome using Barrnap v.0.9. Extracted sequences were entered into megaBLAST searches against the non redundant nucleotide collection in Genbank. The results of this search were used in addition to morphological observation to classify each cell with as much specificity as possible. For each infected cell, best sequences were selected for both host and parasite. All sequences, as well as published core dinoflagellate and additional perkinsid sequences, were added to the 18S alignment from Holt et al. [15] and aligned using MAFFT v.7.481 [16]. The alignment was trimmed in trimAl v.3 (-gt 0.3) [17] and a tree was inferred in IQ-TREE v1.6.12 (model: GTR+F+I+G4) [18]. Four hypervariable V4 region sequences and one V9 region sequence (from samples ERR3446496 and ERR3446502, respectively; [19]) were also included in this analysis, retrieved in a BlastN search against global environmental benthic sequence databases (113,655,329 sequences from 26 studies in total [15]) using our perkinsid parasite sequence as query.

A multi-gene phylogenomic analysis was performed that included the individual host and parasite transcriptomes of infected cells. Curated alignments of 263 conserved genes [20] were queried against each transcriptome in a series of BlastP searches. Hits were aligned to their corresponding curated alignment with MAFFT-LINSI and trimmed in trimAl (-gt 0.8). Trees were constructed for each alignment in IQ-TREE (model: LG+G) so that paralogs, isoforms, and additional contaminant sequences could be eliminated with visual inspection. Transcripts from infected cells were determined to belong to the host or parasite based on their placement in the tree and proximity to closely related lineages. For many genes, host and parasite transcripts were both present, each branching with transcripts from closely related free-living and parasitic lineages, respectively (Table S2). For most infected host cells, single cell or culture derived transcriptomes of healthy individuals of the same species were included for reference. If transcripts from an infected cell were at all ambiguous (a rare occurrence), they were omitted from the analysis. After cleaning, alignments retained at most two representative sequences (if transcripts for both host and parasite were present) from each single cell transcriptome (Table S2). Using SCaFoS v4.55 [21], alignments were parsed to include only relevant taxa (116 in total). A maximum likelihood (ML) phylogeny was inferred using IQ-TREE from a final concatenated alignment of 192 genes (present in ≥60% of included taxa), with 1000 ultrafast bootstraps and the model LG+C60+F+G4 [22]. A second tree was generated for comparison using the same alignment, with 100 nonparametric bootstraps and the model LG+C60+F+G4 PMSF [23].

**References**

1. Cooney EC, Holt CC, Jacko-reynolds VKL, Brian S, Keeling PJ. Photosystems in the eye-like organelles of heterotrophic warnowiid dinoflagellates. *Curr Biol* 2023; 1–9.

2. Cooney EC, Leander BS, Keeling PJ. Phylogenomics shows unique traits in Noctilucales are derived rather than ancestral. *PNAS Nexus* 2022; **1**: 1–11.

3. Cooney EC, Holt CC, Hehenberger E, Adams JA, Leander BS, Keeling PJ. Investigation of heterotrophs reveals new insights in dinoflagellate evolution. *Mol Phylogenet Evol* 2024; **196**: 1–14.

4. Cooney EC, Jacobson DM, Wolfe G V., Bright KJ, Saldarriaga JF, Keeling PJ, et al. Morphology, behavior, and phylogenomics of *Oxytoxum lohmannii*, Dinoflagellata. *J Eukaryot Microbiol* 2024; 1–12.

5. Picelli S, Faridani OR, Björklund ÅK, Winberg G, Sagasser S, Sandberg R. Full-length RNA-seq from single cells using Smart-seq2. *Nat Protoc* 2014; **9**: 171–181.

6. Kolisko M, Boscaro V, Burki F, Lynn DH, Keeling PJ. Single-cell transcriptomics for microbial eukaryotes. *Curr Biol* 2014; **24**: R1081–R1082.

7. Martin M. Cutadapt removes adapter sequences from high-throughput sequencing reads. *EMBnet.journal* . 2011. , **17**: 10–12

8. Bankevich A, Nurk S, Antipov D, Gurevich AA, Dvorkin M, Kulikov AS, et al. SPAdes: A new genome assembly algorithm and its applications to single-cell sequencing. *J Comput Biol* 2012; **19**: 455–477.

9. UniProt: The universal protein knowledgebase in 2021. *Nucleic Acids Res* 2021; **49**: D480–D489.

10. Altschul SF, Gish W, Miller W, Myers EW, Lipman DJ. Basic local alignment search tool. *J Mol Biol* 1990; **215**: 403–410.

11. Laetsch DR, Blaxter ML, Leggett RM. BlobTools : Interrogation of genome assemblies. *F1000Research* 2017; **6**: 1–16.

12. Haas BJ, Papanicolaou A, Yassour M, Grabherr M, Blood PD, Bowden J, et al. De novo transcript sequence reconstruction from RNA-seq using the Trinity platform for reference generation and analysis. *Nat Protoc* 2013; **8**: 1494–1512.

13. Poux S, Arighi CN, Magrane M, Bateman A, Wei CH, Lu Z, et al. On expert curation and scalability: UniProtKB/Swiss-Prot as a case study. *Bioinformatics* 2017; **33**: 3454–3460.

14. Manni M, Berkeley MR, Seppey M, Simão FA, Zdobnov EM. BUSCO Update: Novel and streamlined workflows along with broader and deeper phylogenetic coverage for scoring of eukaryotic, prokaryotic, and viral genomes. *Mol Biol Evol* 2021; **38**: 4647–4654.

15. Holt CC, Hehenberger E, Tikhonenkov D V., Jacko-Reynolds VKL, Okamoto N, Cooney EC, et al. Multiple parallel origins of parasitic Marine Alveolates. *Nat Commun* 2023; **14**: 1–14.

16. Katoh K, Standley DM. MAFFT multiple sequence alignment software version 7: Improvements in performance and usability. *Mol Biol Evol* 2013; **30**: 772–780.

17. Capella-Gutiérrez S, Silla-Martínez JM, Gabaldón T. trimAl: A tool for automated alignment trimming in large-scale phylogenetic analyses. *Bioinformatics* 2009; **25**: 1972–1973.

18. Nguyen LT, Schmidt HA, Von Haeseler A, Minh BQ. IQ-TREE: A fast and effective stochastic algorithm for estimating maximum-likelihood phylogenies. *Mol Biol Evol* 2015; **32**: 268–274.

19. Holman LE, de Bruyn M, Creer S, Carvalho G, Robidart J, Rius M. Detection of introduced and resident marine species using environmental DNA metabarcoding of sediment and water. *Sci Rep* 2019; **9**: 1–10.

20. Burki F, Kaplan M, Tikhonenkov D V., Zlatogursky V, Minh BQ, Radaykina L V., et al. Untangling the early diversification of eukaryotes: a phylogenomic study of the evolutionary origins of Centrohelida, Haptophyta and Cryptista. *Proc R Soc B Biol Sci* 2016; **283**: 1–10.

21. Roure B, Rodriguez-Ezpeleta N, Philippe H. SCaFoS: A tool for selection, concatenation and fusion of sequences for phylogenomics. *BMC Evol Biol* 2007; **7**: 1–12.

22. Quang LS, Gascuel O, Lartillot N. Empirical profile mixture models for phylogenetic reconstruction. *Bioinformatics* 2008; **24**: 2317–2323.

23. Wang H-C, Minh BQ, Susko E, Roger AJ. Modeling site heterogeneity with posterior mean site frequency profiles accelerates accurate phylogenomic estimation. *Syst Biol* 2018; **67**: 216–235.
