# Supplementary material for: Hidden syndinian and perkinsid infections in dinoflagellate hosts revealed by single-cell transcriptomics
Source: ISME J. 2024 Sep 26;18(1):wrae188. doi: 10.1093/ismejo/wrae188 (PMC11468006; doi:10.1093/ismejo/wrae188)
Supplement: Table_S1_wrae188 [file table_s1_wrae188.docx]

**Table S1.** Collection information for isolated single cells. Bar graph shows BUSCO completeness estimates for each transcriptome (compared against the alveolate_odb10 database), containing all host and parasite transcripts. CS = complete buscos, single copy; CD = complete buscos, duplicated copies; F = fragmented; M = missing.
