# Supplementary material for: Hidden syndinian and perkinsid infections in dinoflagellate hosts revealed by single-cell transcriptomics
Source: ISME J. 2024 Sep 26;18(1):wrae188. doi: 10.1093/ismejo/wrae188 (PMC11468006; doi:10.1093/ismejo/wrae188)
Supplement: Table_S2_wrae188 [file table_s2_wrae188.docx]

**Table S2.** Host and parasite transcript presence in datasets used for analysis. Bar graphs show the percentages of present (P; blue) vs. missing (M; pink) transcripts in the greater set of 263 conserved genes, and in the subset of 192 genes ultimately used in the phylogenomic analysis (those represented by ≥60% of OTUs in the final tree). Hosts are highlighted in grey, parasites are unhighlighted.
